# Recent insights into metabolic and signalling events of directional root growth regulation and its implications for sustainable crop production systems

**DOI:** 10.3389/fpls.2023.1154088

**Published:** 2023-03-16

**Authors:** Katarzyna Retzer, Wolfram Weckwerth

**Affiliations:** ^1^ Laboratory of Hormonal Regulations in Plants, Institute of Experimental Botany, Czech Academy of Sciences, Prague, Czechia; ^2^ Department of Functional and Evolutionary Ecology, Faculty of Life Sciences, Molecular Systems Biology (MoSys), University of Vienna, Wien, Austria; ^3^ Vienna Metabolomics Center (VIME), University of Vienna, Wien, Austria

**Keywords:** TOR, SnRK1, SnRK2, ABA, auxin, green systems biology, panomics, metabolomics

## Abstract

Roots are sensors evolved to simultaneously respond to manifold signals, which allow the plant to survive. Root growth responses, including the modulation of directional root growth, were shown to be differently regulated when the root is exposed to a combination of exogenous stimuli compared to an individual stress trigger. Several studies pointed especially to the impact of the negative phototropic response of roots, which interferes with the adaptation of directional root growth upon additional gravitropic, halotropic or mechanical triggers. This review will provide a general overview of known cellular, molecular and signalling mechanisms involved in directional root growth regulation upon exogenous stimuli. Furthermore, we summarise recent experimental approaches to dissect which root growth responses are regulated upon which individual trigger. Finally, we provide a general overview of how to implement the knowledge gained to improve plant breeding.

## 1 Introduction

Roots evolved to grow into the soil, sense environmental changes and adjust their architecture and growth direction to maximize water and nutrient uptake and avoid obstacles and toxic compounds (De Pessemier et al., 2013; Pierik and Testerink, 2014; Kolb et al., 2017; Gupta et al., 2020; Zhang and Friml, 2020; D E Lima et al., 2021; Karlova et al., 2021; Leftley et al., 2021; González-García et al., 2022). As the so-called hidden half of the plant, roots are not easy to phenotype under natural growth conditions (Piñeros et al., 2016; Atkinson et al., 2019). Very informative but methodological demanding is to track root growth in soil under controlled conditions, which includes root visualization *via* X-ray computed tomography (X-ray CT) or other imaging techniques (Mairhofer et al., 2013; Mairhofer et al., 2017; Atkinson et al., 2019; Handakumbura et al., 2021; Doussan, 2022). Evaluation of root traits in 3D, including root length, the outgrowth of lateral roots, and overall response to soil density, allow the studying of fine modulation of root growth plasticity directly in soil (Rogers et al., 2016; Atkinson et al., 2019; Freschet et al., 2021). Growing roots under controlled conditions in soil also allows to perform analytical studies after the application of defined stress treatments or changes in nutrient composition, to obtain detailed information about changes in the transcriptome, proteome, phosphoproteome, metabolome, root exudates, or root-microbe interaction (Weckwerth, 2008; Weckwerth, 2011; Ghatak et al., 2016; Ghatak et al., 2017; Chen and Weckwerth, 2020; Ghatak et al., 2020; Weckwerth et al., 2020; Ghatak et al., 2021; Ghatak et al., 2022a). On the other hand, tracking of immediate adaptation of directional root growth, root morphological changes, or protein trafficking is not possible when the root is hidden in the soil and requires depending on the studied trigger individual experimental setups (Downie et al., 2012; Yokawa et al., 2014a; Ma et al., 2019; Lacek et al., 2021; Taylor et al., 2021; Mehra et al., 2022). Recently, the impact of direct root illumination on root growth plasticity and function and seedlings with roots exposed to light and shaded were comprehensively studied (Xu et al., 2013; Silva-Navas et al., 2015; Silva-Navas et al., 2016; Silva-Navas et al., 2019; Li et al., 2020; García-González et al., 2021a; García-González et al., 2021b; Lacek et al., 2021; Cabrera et al., 2022; García-González et al., 2022). Seedlings grown on agar-supplemented growth medium can be grown with roots shaded from direct illumination, and the commercially available so-called D-root system is used by several laboratories (Silva-Navas et al., 2015). The D-root system enables growing roots in controlled laboratory conditions, shaded from direct illumination, and keeping the shoot illuminated, which reduces plant stress (Silva-Navas et al., 2015; Lacek et al., 2021; Cabrera et al., 2022). Direct root illumination diminishes nutrient uptake and distribution, alters root system architecture and shoot-root communication, and results in retained root growth adaptation upon additive stress exposure like salt and osmotic pressure (Silva-Navas et al., 2015; Cabrera et al., 2022). Furthermore, direct root illumination significantly enhances root growth deviation from vertical, especially when shootward auxin transport is impaired (García-González et al., 2021a; Lacek et al., 2021; García-González et al., 2022). Moreover, direct root illumination delimits the ability to respond to unilateral salt stress efficiently, negative halotropism, and towards higher water gradient, positive hydrotropism (Yokawa et al., 2014a; Wan et al., 2019; Li et al., 2020). This review will discuss these additive stress responses and involved metabolic and highly conserved signalling pathways controlling directional root growth and metabolism. These processes are decisive for the root belowground performance, determine the shoot-root communication and have substantial implications for understanding plant productivity and performance under harsh environmental conditions. Consequently, it is necessary to study these phenomena to derive essential plant breeding strategies and develop a sustainable agricultural process based on a high diversity of germplasm collections (Weckwerth, 2011; Ghatak et al., 2022a). We will discuss this in the last chapter.

## 2 Root tropism and directional root growth

**Figure 1:**
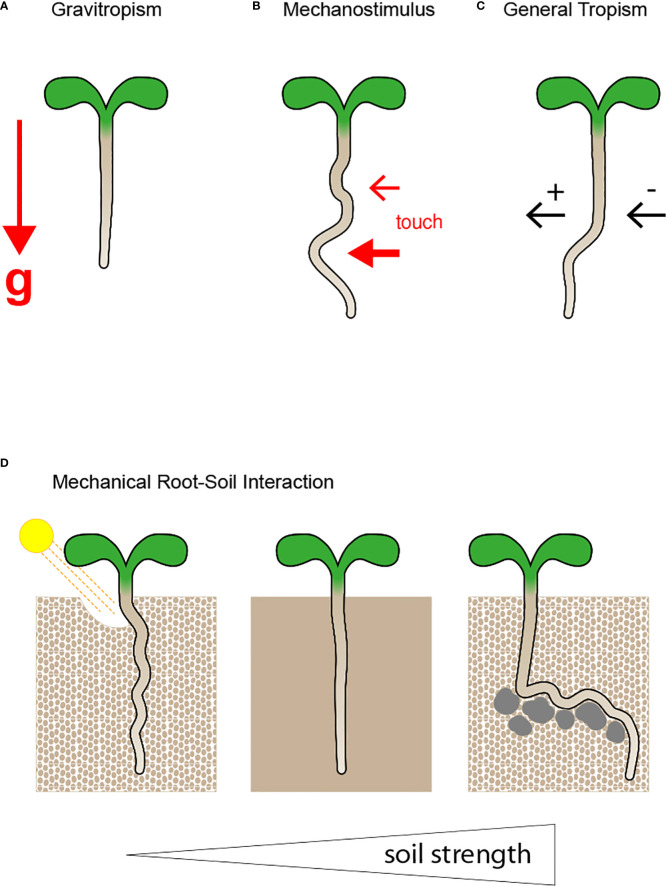
Root growth direction and pattern are modulated by manifold stimuli. **(A)** Roots evolved to steer growth direction along the gravity vector (g), as indicated by the red arrow. Although gravity is a continuous and constant predominant force, the soil is a heterogeneous mixture, and other exogenous stimuli result in root growth deviation. **(B)** Roots are exposed to diverse mechanical/touch stimuli,indicated by the red arrows, depending on the growth medium strength, and their response to the mechanical pressure defines root growth pattern. **(C)** The soil composition is variable, and nutrients, water, indicated by the black arrows, and harmful substances are randomly distributed, wherefore the root developed to grow towards (positive tropism) or away (negative tropism) from distinct stimuli. **(D)** Soil can be either loose, compact or exhibit impenetrable obstacles. Roots must adjust their root growth behaviour accordingly to efficiently explore their surroundings and ensure nutrient uptake and plant productivity. The root adjusts root growth behaviour to reduce soil strength. In loose soil the root is circumnutating, whereby it moves light particles aside while manoeuvring through the soil. When the soil becomes more compact the root needs to change root properties to grow through dense areas by changing its mechanical properties of the very root tip, which enhance the efficiency of soil penetration.

The ability of plants to adjust the direction of their growth allows their survival in a continuously changing environment (**Figures 1A–C**) (Pierik and Testerink, 2014; Lacek et al., 2021). Roots evolved as underground organs embedded in the soil, a heterogeneous mixture regarding nutrient composition and structure, which results in unilateral stimuli that can change the growth direction of the root tip, also known as tropism (**Figures 1C, D**) (Kolb et al., 2017; Silva-Navas et al., 2019; Muthert et al., 2020). Altogether, roots of higher plants are susceptible to their environment and continuously perceive signals, including information about gradients of nutrients and water, changing temperature and light, and the occurrence of toxins and pathogens. All exogenous stimuli activate the root interwoven signalling cascades that modulate growth speed and direction (Barrada et al., 2015; Miotto et al., 2021; Pierik et al., 2021; Retzer and Weckwerth, 2021; van Gelderen et al., 2021). Although many molecular key players have been described over the decades, a comprehensive understanding of how directional root growth is orchestrated under different growth conditions remains elusive (Thompson and Holbrook, 2004; Migliaccio et al., 2009; Wyatt and Kiss, 2013; Lopez et al., 2014; Retzer et al., 2014; Vandenbrink and Kiss, 2019; Muthert et al., 2020).

### 2.1 Direct root illumination results in growth deviation and impairment as stress reaction

Besides root gravitropism, the impact of direct root illumination is currently studied intensively, and the interplay between gravitropism and phototropism of the root was recently extensively reviewed by Cabrera et al. (2022) and Lacek et al. (2021), therefore we will only highlight the results and experimental setups used in the key studies (Xu et al., 2013; Yokawa et al., 2014a; Silva-Navas et al., 2015; Silva-Navas et al., 2016; Silva-Navas et al., 2019). Photoreceptors are expressed along the root, whereby blue light receptors are located at the transition and elongation zone, and red light receptors in the meristematic zone, which leads to different root growth responses depending on the wavelength a root is exposed to (Dyachok et al., 2011; Mo et al., 2015; Silva-Navas et al., 2015). Blue light perception in the elongation zone modulates among others the abundance of the auxin efflux carrier PIN-FORMED 2 (PIN2) at the plasma membrane (PM), by orchestrating its phosphorylation status, which was associated with PIN2 stabilization at the PM and reduced endocytosis and transport towards the lytic vacuole for its degradation (Laxmi et al., 2008; Wan et al., 2012; Retzer et al., 2019). Furthermore, direct root illumination with blue light correlated with an enhanced elongation rate of the root and was therefore named ‘light escape mechanism’ (Yokawa et al., 2014a; Silva-Navas et al., 2016; Yokawa and Baluška, 2016; Yokawa et al., 2016). Several studies investigated the putative molecular mechanisms that orchestrate negative root phototropism, and similar to other responses, an intact and flexible cytoskeleton is fundamental, and actin-filament bundling was shown to support root growth away from the light source (Dyachok et al., 2011; García-González and van Gelderen, 2021).

The root experiences direct root illumination as stress, and responds with elevated REACTIVE OXYGEN SPECIES (ROS) levels (Yokawa et al., 2014a; Yokawa et al., 2014b; Silva-Navas et al., 2015; Yokawa et al., 2016). ROS accumulation in the meristematic zone is associated with a reduced proliferation rate and, consequently, reduced total root length (Yokawa et al., 2014b; Yokawa et al., 2016). On the other hand, illumination of the root at the position of the transition zone and early differentiation zone stimulates cell elongation (Silva-Navas et al., 2016). To counterbalance the light-induced ROS production, flavonoid synthesis is upregulated to act as scavengers, which alters additionally cellular responses of illuminated roots (Silva-Navas et al., 2016; Cabrera et al., 2022). Under unilateral illumination with blue light, cells closer to the light source accumulated flavonoids, and asymmetric cell elongation led to root bending movement away from the light (Silva-Navas et al., 2016).

Plant responses to enhanced illumination are also overlapping with temperature sensing, and also elevated temperatures result in root growth arrest and delimited ability to expand root surface, which correlates with reduced nutrient uptake (Calleja-Cabrera et al., 2020; Ghatak et al., 2020; Kim et al., 2020; D E Lima et al., 2021; González-García et al., 2022). To study thermal-related stress responses of roots a novel device was recently introduced by González-García et al. (2022), namely the TGRooZ device that allows to apply temperature gradients to roots grown on agar supplemented medium and additionally shade them from direct illumination (González-García et al., 2022). Even if the air is heated up, below ground temperatures drop gradually when the root grows deeper into the soil, which allows efficient root system architecture establishment compared to plants with shoots and roots exposed to the same elevated temperature (González-García et al., 2022). Furthermore, the comparison of roots grown in constant heat to those grown along a temperature gradient in the TGRooZ device showed that constant heat stress applied to the root reduces especially phosphate uptake and also delimits beneficial bacterial community assembly (González-García et al., 2022). Grafting experiments demonstrated the importance of controlled root-soil interaction, which attracts beneficial microbes and delimits pathogen accumulation (Cardarelli et al., 2020; Muramoto et al., 2022). How plant–microbe interactions improve crop productivity and plant health is currently intensively studied, whereby root exudate secretion plays an essential role in the regulation of the rhizosphere microbiome composition, which is in more detail discussed in chapter 5 (Handakumbura et al., 2021; Ghatak et al., 2022a).

### 2.2 Root gravitropism and response to mechanostimulus are challenging to dissect

Konstantinova et al. (2021), thoroughly reviewed current knowledge around root gravitropic regulation (Konstantinova et al., 2021). Early studies of auxin transporter mutants that were selected because of their agravitropic roots indicated an essential role of auxin for directional root growth adaptation, which was confirmed in manifold follow-up studies (Okada and Shimura, 1990; Luschnig et al., 1998; Swarup et al., 2005; Abas et al., 2006; Retzer et al., 2017; Retzer et al., 2019). Further experimental evidence for the role of auxin during root growth adaptation was delivered by visualization of asymmetric auxin gradients during the gravitropic response in the epidermal cells of the root tip, which are lost in the auxin transporter mutants (Friml et al., 2002; Ottenschläger et al., 2003; Abas et al., 2006; Wisniewska et al., 2006; Swarup and Bennett, 2009). Beside auxin gradients, modulation of signalling waves along the root tip of other messenger molecules was investigated. Models including calcium spikes and pH changes along the root tip are currently discussed to initiate immediate root growth changes through environmental stimuli, and intensive studies are ongoing to dissect the order of molecular events (Cole and Fowler, 2006; Barbez et al., 2017; Fendrych et al., 2018; Dubey et al., 2021; Li et al., 2021).

Gravitational biology is a part of the so-called mechanobiology (van Loon, 2009). According to van Loon (2009), there is no biochemical adaptation without prior mechanical change (van Loon, 2009). In the case of roots responding to gravitropism it is suggested that asymmetric auxin distribution results in asymmetric cell expansion, which changes the root growth path by modulation of mechanical properties of individual cells (Konstantinova et al., 2021; Jonsson et al., 2022). Among the first publications describing root tropisms, gravitropism was suggested as a modification of an ancestral plant mechanical sensing system (Trewavas and Knight, 1994). Furthermore, earlier studies of directional root growth were dedicated to distinguishing responses to gravistimulus and mechanostimulus. It became apparent that the gravitropic response and adaptation to mechanical or touch stimulus are challenging to dissect (**Figures 1A, B**) (Massa and Gilroy, 2003; Najrana and Sanchez-Esteban, 2016; Yan et al., 2018; Tojo et al., 2021). It requires exposing plants to microgravity in special centrifuges on earth or by performing experiments during spaceflights (Vandenbrink et al., 2014; Najrana and Sanchez-Esteban, 2016; Muthert et al., 2020; Chin and Blancaflor, 2022; Herranz et al., 2022). Experimental setups that combined the testing of responses to direct root illumination under microgravity, showed that reduced gravity led to a decrease of meristem activity, as well altered phototropic responses, which is reversible when gravity is re-established (Valbuena et al., 2018; Herranz et al., 2019; Villacampa et al., 2022). RNAseq and metabolomic analysis of plants exposed to microgravity in combination with direct root illumination further confirmed the importance of cytoskeleton filament bundling, membrane and cell wall reorganization for efficient tropistic responses (Millar and Kiss, 2013; Herranz et al., 2019; Shymanovich et al., 2022).

### 2.3 Hydrotropism and halotropism are linked to the cellular stress sensor SnRK2

Due to their sessile lifestyle plants evolved regulatory kinase families, SNF1-RELATED PROTEIN KINASE 2 and 3 (SnRK2 and 3), which consist of multiple members, whereby the abscisic acid (ABA) sensitive SnRK2.2, SnRK2.3 and SnRK2.6 were manifold linked to directional root growth control (Kulik et al., 2011; Crepin and Rolland, 2019; Yang et al., 2019). ABA signalling is crucial to modulate plant growth and function under osmotic stress and drought, and also root growth deviation during hydrotropic and halotropic responses (Dietrich et al., 2017; Belda-Palazón et al., 2020; Kawa et al., 2020; Karlova et al., 2021; Belda-Palazón et al., 2022).

Dietrich et al. (2017), showed that SnRK2.2 expression in the root cortex is sufficient to drive directional root growth towards higher water potential, which corresponds to the crucial role of abscisic acid (ABA) dependent SnRK2s that underpin root growth adjustment under drought (Dietrich et al., 2017; Belda-Palazón et al., 2020; Belda-Palazón et al., 2022; Mehra et al., 2022). Hydrotropism was considered as an active form of directional root growth regulation to initiate drought avoidance and is known to be affected by gravity and direct root illumination (Miyazawa et al., 2011; Iwata et al., 2013). So far it was shown that ABA and cytokinins fundamentally modulate root responses to steer hydrotropic root growth, which counteracts other exogenous stimuli, including gravity, light or touch (Cassab et al., 2013; Li et al., 2020). Importantly, direct root illumination interferes negatively with root hydrotropism, whereby transcriptomic analysis indicates decreased expression of genes involved in starch metabolism in the root tip of dark-grown roots (Li et al., 2020). This implies that hydrotropism counteracts gravitropism by decreasing amyloplast content in the columella, but further investigations are required to prove this hypothesis (Li et al., 2020).

Negative root halotropism was also linked to the action of ABA-sensitive SnRK2 orchestrated signalling pathways, whereby SnRK2.6 was found to phosphorylate a microtubule-associated protein, SP2L, which results in anisotropic cell expansion and root twisting in the transition zone (Yu et al., 2022). Also, halotropism was shown to be slower in illuminated roots than shaded roots, but molecular cues are still elusive (Yokawa et al., 2014a). It is known that SnRK2 act at the cross-road of developmental and stress adaptation processes, and they interconnect not only signalling pathways modulated by different phytohormones, but also those dependent on signalling molecules like ROS or Ca^2+^ and energy homeostasis (Yang and Guo, 2018; Crepin and Rolland, 2019; Jamsheer, 2019; Signorelli et al., 2019; Yang et al., 2019).

## 3 Evolutionary conserved cellular sensors coordinate plant stress responses

**Figure 2:**
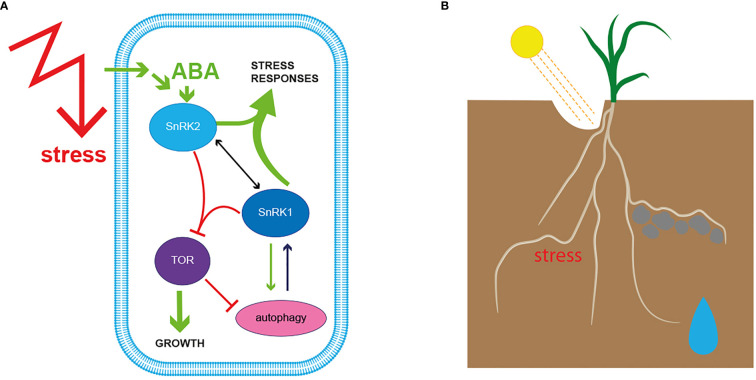
**(A)** Intracellular molecular sensors evaluate perceived signals against available resources, resulting in cellular remodelling to balance plant growth versus stress responses. Mechanical and biochemical responses at the cellular level orchestrate plant shape and function and modulation of cell expansion during tropistic responses. Exogenous stimuli generate signals that are transmitted by signalling molecules inside the cell, where a set of kinases, known as molecular gatekeepers, steer the responses. Under beneficial growth conditions TARGET OF RAPAMYCIN (TOR) positively regulates plant growth processes. When energy, in the form of sugars or other nutrients is delimited, SNF1-RELATED PROTEIN KINASE 1 (SnRK1) represses TOR activity to reorganize resource availability in the cell, which may also include enhancement of autophagy and alter cell activity to counteract the stress trigger. Individual environmental signals activate kinases from the SnRK2 family, which results in a tight interplay with SnRK1 to delimit TOR activity further. ABA-activated SnRK2s initiate cellular events that allow the plant to cope with harsh environmental conditions like drought and further regulate root hydrotropism (described under 2.3). **(B)** The soil is a heterogenous mixture, regarding its nutrient composition as well as its mechanical properties, therefore roots evolved to respond to manifold exogenous signals simultaneously to adjust growth direction and surface enlargement. Roots grow away from harmful conditions and around obstacles they cannot penetrate, but adjust root growth direction towards nutrients and water. TOR-SnRK1-SnRK2 interaction modulates the adjustment of root growth to a plethora of simultaneously occurring signals to ensure efficient plant growth. The complexity of their interplay is described in chapter 3.

Plants, like all organisms, regulate resource and energy homeostasis at the cellular level *via* two evolutionary conserved protein complexes, namely TARGET OF RAPAMYCIN (TOR) and SnRK1 (Mair et al., 2015; Nukarinen et al., 2016; Roustan et al., 2016; Jamsheer, 2019; Margalha et al., 2019; Ryabova et al., 2019; Fichtner et al., 2020; Jamsheer et al., 2021). The subunit composition of both complexes and their interactions with each other were thoroughly discussed in recent publications. Therefore, we will briefly summarize in this review the importance of both complexes for stress growth adaptation (Henriques et al., 2014; Broeckx et al., 2016; Margalha et al., 2016; Roustan et al., 2016; Salem et al., 2018; Jamsheer, 2019; Retzer and Weckwerth, 2021). Plants are divided into the aboveground-located shoot and the belowground-located root. The shoot generates energy in the form of carbohydrates over photosynthesis, wherefore its productivity primarily depends on light quality and intensity (Pierik and Testerink, 2014; Fichtner et al., 2020; Miotto et al., 2021; Retzer and Weckwerth, 2021). Furthermore, shoot activity also depends on the delivery of water and nutrients acquired from the root (Grierson et al., 2014; Wu et al., 2019; Karlova et al., 2021; Retzer and Weckwerth, 2021; Ghatak et al., 2022a). The shoot and the root exchange signals and compounds constantly to ensure healthy growth and coordinated reorganization of plant architecture. TOR and SnRK1 play a crucial role at the cellular and subcellular levels to ensure efficient plant shape and function adjustments to exogenous and intrinsic signals (Pérez-Pérez et al., 2010; Mair et al., 2015; Pedrotti et al., 2018; Wurzinger et al., 2018; Fürtauer et al., 2019). TOR orchestrates plant development and growth processes under favourable growth conditions, whereas SnRK1 inhibits TOR action when energy and resource levels are low, or other stresses endanger plant growth (**Figure 2A**) (Nukarinen et al., 2016; Crepin and Rolland, 2019; Ryabova et al., 2019; Retzer and Weckwerth, 2021).

SnRK1 acts as a central hub on the cellular level to merge information about energy, nutrient and stress at the whole plant level and activates signalling pathways to adapt transcriptome, proteome and metabolome to maintain cellular mechanisms when resources are limited (Baena-González et al., 2007; Nukarinen et al., 2016; Weiste et al., 2017; Ramon et al., 2019; Belda-Palazón et al., 2022; Van Leene et al., 2022; Son et al., 2023). As demonstrated by Nukarinen et al., 2016 (Nukarinen et al., 2016) and other studies, SnRK1 is well known for its TOR-antagonistic role of coordinating shoot function, shoot-root communication and metabolomic balance under less favourable growth conditions (Nukarinen et al., 2016; Ramon et al., 2019; Van Leene et al., 2019; Sun et al., 2021; Belda-Palazón et al., 2022). Overall, SnRK1 activity is required to regulate transcriptional and translational cellular profile switches, among others, by fine-tuning the availability and signalling of phytohormones (Baena-González and Hanson, 2017; Jamsheer et al., 2021; Belda-Palazón et al., 2022). Furthermore, SnRK1 orchestrates resources breakdown of existing resources, like starch by amylases or other cellular components by autophagy (Baena-González et al., 2007; Signorelli et al., 2019). Overexpression of the catalytic subunit of SnRK1 results in increased autophagy in the root epidermis (Batoko et al., 2017; Soto-Burgos and Bassham, 2017). Initiation and modulation of autophagy in plants are under the tight control of both cellular energy censors, SnRK1 and TOR, whereby TOR itself is inhibited by SnRK1 when cellular re-arrangement is required. TOR activity inhibition is enhanced when environmental signals are additionally perceived and transmitted by SnRK2-dependent pathways (**Figure 2B**) (Pu et al., 2017; Huang et al., 2019; Henriques et al., 2022). The complex interactions of SnRK1, SnRK2, TOR and autophagy that maintain cellular function under different growth conditions, which underpins plant growth and fitness, are currently intensively studied and will provide a better understanding of the environmental impact on plant productivity (Hill et al., 2013; Barrada et al., 2015; Li et al., 2018; Ghatak et al., 2022a; Kashkan et al., 2022).

## 4 Dissection of interactions of metabolomic key-regulators in dark-grown roots is the next step to understanding individual root growth responses to exogenous stimuli

Millar and Kiss (2013), studied metabolomic responses during different tropistic responses, and showed that certain metabolomic pathways are differently regulated compared to unstimulated growth (Millar and Kiss, 2013). The upregulation DARK INDUCIBLE 6 (ASN1/DIN6), which is an established marker to track the activity of the central cellular energy sensor SNF1-RELATED PROTEIN KINASE 1 (SnRK1), during gravitropic and phototropic responses is highly interesting, as well the downregulation of the CHALCONE SYNTHASE (ATCHS/CHS/TT4) that is a key enzyme involved in the biosynthesis of flavonoids (Baena-González et al., 2007; Millar and Kiss, 2013; Retzer and Weckwerth, 2021; Peixoto and Baena-González, 2022). Integrating multiple signalling pathways triggered by exogenous stimuli requires the interplay of molecular sensors at the cellular level (Nukarinen et al., 2016; Wurzinger et al., 2017; Retzer and Weckwerth, 2021). Cellular mechanical and biochemical responses are modulated differently under changing growth conditions, which define the availability of resources and energy (van Loon, 2009; Clark et al., 2021; Lacek et al., 2021; Retzer and Weckwerth, 2021; Aronne et al., 2022; Montes et al., 2022). The combination of roots shaded from direct illumination, by using the D-root system, with simulated microgravity conditions showed that root illumination enhances root growth deviation more, compared to roots grown shaded from light (Villacampa et al., 2022). Villacampa et al. (2022), showed that mutants with reduced flavonoid synthesis display even more enhanced randomness of directional root growth when they experience microgravity (Villacampa et al., 2022). These results correlate with experiments performed in the lab at earth gravity conditions (1g) by Silva-Navas et al. (2016), showing how different local flavonoid and ROS accumulation occur in roots exposed to different illumination regimes (Silva-Navas et al., 2016). The role of flavonoids, which belong to polyphenolic secondary metabolites, in crosstalk with ROS signalling in distinct root zones was previously discussed intensively in several studies and reviews, and it is obvious that their abundance, distribution and function is extremely variable depending on growth conditions (Yokawa et al., 2014b; Lacek et al., 2021; Cabrera et al., 2022). Furthermore, the complex interaction of flavonoid and ROS signalling with other pathways, especially those including phytohormones, is heavily investigated and not without controversial outcomes, which again demonstrates the complexity of plant responses to the sum of experienced exogenous stimuli (Lacek et al., 2021).

## 5 How important are metabolic and signalling control events in root tropism for plant productivity, resilience and sustainable crop production systems?

Plants possess rapid root growth regulation to manoeuvre efficiently through heterogenous soil upon manifold, simultaneously occuring exogenous triggers, and this is of high agronomical importance. Growth conditions modulate plant traits, including metabolism. Recently modulation of plant growth metabolism was described as a desirable target to ensure sustainable agriculture (Li et al., 2018). The root–shoot communication system controls processes like mineral and nutritional supply, grain filling, biotic and abiotic stress resistance and is decisive for plant productivity and crop production systems. Highly conserved metabolic and signalling pathways play a crucial role (Li et al., 2017; Chen and Weckwerth, 2020). Root growth depends on sugars transported from the shoot, whereby under drought conditions, SnRK2.2 enhances sugar transport from the shoot towards the root by enhancing transport activity *via* phosphorylation of SWEET11 to promote root branching (Chen et al., 2022). This is favourable when photosynthesis is highly active, whereas when the plant’s energy resources are depleted, SnRK1 was reported to adjust sugar metabolism to maintain root branching (Muralidhara et al., 2021). Furthermore, primary root meristem activity is repressed under drought stress, where ABA-sensitive SnRK2.2 and SnRK2.3 induce subcellular re-localization of the catalytic subunit of SnRK1 to inhibit TOR activity (Belda-Palazón et al., 2022). A wider root system results in a higher probability for the plant to acquire water, and nutrients, including nitrogen (van Gelderen et al., 2021). The tight modulation of shoot-root communication to balance cellular C/N metabolism is highly important for plant fitness and productivity (Weckwerth, 2003; Fürtauer et al., 2019). C/N metabolism is furthermore closely coupled to energy and resource regulation at the cellular level, and a more detailed understanding of the distribution of sugars and nutrients between shoot and root under different cultivation conditions will further result in more efficient plant cultivation (Nukarinen et al., 2016; Retzer and Weckwerth, 2021). Recent studies have demonstrated a tight regulation of nutrient availability and TOR regulation in the root system, which controls the C/N/S availability for the shoot (Dong et al., 2017; Dong et al., 2022). Another important aspect for plant growth and productivity is the root–soil microbiome interactions (Ghatak et al., 2022a). The soil microbiome contributes strongly to plant growth and productivity (Ghatak et al., 2022a). At the same time, plants can control the rhizosphere microbiome by root exudates, exhibiting biological nitrification inhibition activity on the bacterial and archaeal nitrifyer community and thereby controlling the nitrogen availability for the plant (Ghatak et al., 2022a). This plant-microbe-soil interaction has strong implications for sustainable crop production systems with respect to excessive nitrogen fertilizer application to the field (Ghatak et al., 2022a). Root architecture plays a decisive role here (Saleem et al., 2018; Ghatak et al., 2022b). In a recent study on the important cereal crop plant pearl millet root exudation was dependent on root architecture and showed at the same time variation under drought stress conditions and different biological nitrification inhibition, thereby influencing the rhizosphere microbiome differently (Saleem et al., 2018; Ghatak et al., 2022b). A recent study investigated signalling processes involved in root exudation and biological nitrification inhibition and identified involvement of ABA and auxin signalling (Ma et al., 2023). As discussed earlier, ABA and auxin signalling are intimately bound to nutrient availability and SnRK1/TOR signalling (Retzer and Weckwerth, 2021). These processes are not predictable from genome sequences and demand more advanced technological platforms such as metabolomics, shotgun proteomics and phosphoproteomics, a PANOMICS platform, integrating all these technologies (Weckwerth, 2008; Weckwerth, 2011; Chen and Weckwerth, 2020; Weckwerth et al., 2020). Most importantly, the PANOMICS platform allows screening for these signalling events in root tropism in large genotyped germplasm collections, providing a new paradigm for marker-assisted plant breeding approaches (Chen and Weckwerth, 2020; Weckwerth et al., 2020; Ghatak et al., 2022a). Germplasm collections of important staple food crops are available, and genotyping of these collections in combination with PANOMICS technologies is the next step into a novel era of plant breeding (Weckwerth et al., 2020). The control of the root system architecture and its directional growth by metabolic and signalling pathways is not predictable by genome sequences of these large germplasm collections alone, phenotypic variance is only predicted for about 40% in these collections (Weckwerth et al., 2020). Therefore a PANOMICS platform will contribute strongly to elucidate these processes in much more detail and provide plant breeders with novel hitherto inaccessible biomarkers for marker-assisted breeding approaches (Weckwerth et al., 2020; Ghatak et al., 2022a).

## Author contributions

KR and WW drafted, wrote and edited the article. All authors contributed to the article and approved the submitted version
